# Heterogeneous local order in self-assembled nanoparticle films revealed by X-ray cross-correlations

**DOI:** 10.1107/S2052252518005407

**Published:** 2018-04-28

**Authors:** Felix Lehmkühler, Florian Schulz, Martin A. Schroer, Lara Frenzel, Holger Lange, Gerhard Grübel

**Affiliations:** a Deutsches Elektronen-Synchrotron (DESY), Notkestrasse 85, 22607 Hamburg, Germany; b The Hamburg Centre for Ultrafast Imaging, Luruper Chaussee 149, 22761 Hamburg, Germany; c Institute of Physical Chemistry, University of Hamburg, Grindelallee 117, 20146 Hamburg, Germany; d European Molecular Biology Laboratory EMBL c/o DESY, Notkestrasse 85, 22607 Hamburg, Germany

**Keywords:** self-assembled nanoparticle films, X-ray cross-correlations, XCCA, gold nanoparticles

## Abstract

We report on the self-assembly of gold nanoparticles coated with a soft poly(ethylene glycol) shell studied by X-ray cross-correlation analysis. Depending on the initial concentration of gold nanoparticles used, structurally heterogeneous films were formed. The films feature hot spots of dominating four- and sixfold local order with patch sizes of a few micrometres, containing 10^4^–10^5^ particles. The amplitude of the order parameters suggested that a minimum sample amount was necessary to form well ordered local structures. Furthermore, the increasing variation in order parameters with sample thickness demonstrated a high degree of structural heterogeneity. This wealth of information cannot be obtained by the conventional microscopy techniques that are commonly used to study nanocrystal superstructures, as illustrated by complementary scanning electron microscopy measurements.

## Abstract 2

An X-ray cross-correlation study of the self-assembly of soft-shell nanoparticles is presented. It is demonstrated that the assembled films are structurally heterogeneous, with dominant hexagonal and cubic symmetries forming patches of 10^4^–10^5^ particles.

## 1. Introduction   

Over recent decades, nanoparticle superstructures have attracted increasing interest in research and technology because of their wide range of potential applications (Talapin *et al.*, 2010 ▸; Song & Cölfen, 2010 ▸; Wang *et al.*, 2013 ▸). For such structures, the desired nanoparticle properties are preserved and they can be integrated into current technology more easily (Reichhelm *et al.*, 2017 ▸). The most commonly used route to obtain nanoparticle superstructures is the self-assembly process. Therefore, the self-assembly of nanoparticles is intensively studied in materials and nanoscience; it promises straightforward production of functional nanostructures, avoiding sophisticated and costly techniques such as high-resolution lithography (Bishop *et al.*, 2009 ▸; Grzelczak *et al.*, 2010 ▸; Luo *et al.*, 2015 ▸; Nie *et al.*, 2009 ▸). Self-assembled structures may demonstrate various properties that differ from bulk material, *e.g.* exceptional mechanical properties (Dreyer *et al.*, 2016 ▸). Thus, there is a fundamental interest in the mechanisms of structure formation on the nanoscale and in new phases, focusing on crystalline structures and the collective properties that might emerge (Boles *et al.*, 2016 ▸; Busseron *et al.*, 2013 ▸; Klinkova *et al.*, 2014 ▸; Ye *et al.*, 2015 ▸). However, structures with only local or mesoscale order are typically obtained as a result of the complexity of the self-assembly process. For such structures, electron microscopy can provide insight into the local order, but with rather poor statistics and limited volume information which is crucial for understanding the relationship between structure and mechanical, optical and electronic properties. Here, scattering methods offer valuable complementary characterization.

In general, scattering methods are widely employed to study the structure of matter on different length scales. Diffraction studies of the structures of assembled colloidal films have been performed for decades (Clark *et al.*, 1983 ▸; Van Winkle & Murray, 1986 ▸), however, the inherent averaging typically allows access to averaged information only, such as particle–particle pair correlation functions 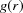. One way to overcome this limitation is the X-ray cross-correlation analysis (XCCA) approach towards diffraction patterns from coherent X-ray scattering experiments. First proposed by Kam 40 years ago (Kam, 1977 ▸), cross-correlation methods are now employed in reconstruction studies of particles in solution (Saldin *et al.*, 2011 ▸; Pedrini *et al.*, 2013 ▸) and considered for obtaining single-particle imaging at free-electron laser (FEL) sources (Starodub *et al.*, 2012 ▸; Kurta *et al.*, 2017 ▸). The possibility of gaining structural information in dense systems such as liquids or glasses was demonstrated in laser scattering experiments in the 1980s (Clark *et al.*, 1983 ▸; Ackerson *et al.*, 1985 ▸) and revived by X-ray studies of colloidal glasses (Wochner *et al.*, 2009 ▸). In recent years, the potential of XCCA to study structures beyond 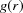 has been investigated by various theories and simulation studies of two-dimensional systems (Altarelli *et al.*, 2010 ▸; Kurta *et al.*, 2012 ▸; Lehmkühler *et al.*, 2014 ▸; Malmerberg *et al.*, 2015 ▸; Latychevskaia *et al.*, 2015 ▸; Martin, 2017 ▸; Lhermitte, Tian *et al.*, 2017 ▸), and shown experimentally for thin colloidal and polymer systems (Schroer *et al.*, 2014 ▸, 2015 ▸; Gutt *et al.*, 2014 ▸; Liu *et al.*, 2017 ▸), liquid crystals (Zaluzhnyy *et al.*, 2016 ▸, 2017 ▸) and colloids and nanocrystals (Mendez *et al.*, 2014 ▸; Mancini *et al.*, 2016 ▸; Lehmkühler *et al.*, 2016 ▸; Schroer, Westermeier *et al.*, 2016 ▸; Zaluzhnyy *et al.*, 2017 ▸). In addition, cross-correlation has recently been demonstrated as a valuable tool for optimizing scattering signals of noisy data (Lhermitte, Stein *et al.*, 2017 ▸).

We previously demonstrated the potential of XCCA combined with scanning X-ray microscopy to probe the local order of colloidal films of thicknesses up to a few micrometres (Schroer *et al.*, 2015 ▸). In the present work, we expand this approach in order to investigate the structure formation of highly monodisperse self-assembling gold nanoparticles whose interaction is dominated by a soft shell based on poly(ethylene glycol) (PEG). We study the degree of local order of self-assembled layers with a spatial resolution of 500 nm, only limited by the size of the probing X-ray beam. We find the formation of heterogeneous structures dominated by patches of four- and sixfold order. The degree of dominating local order increases with sample thickness, indicating that a minimum number of particles are necessary to form well defined ordered patches. In particular, well ordered sixfold patches are found to have a characteristic size of about 3.5 µm, which corresponds to layers of more than 100 × 100 particles.

## 2. Experimental   

### 2.1. Samples   

Gold nanoparticles (AuNP) were synthesized using the seeded-growth protocol presented by Bastús *et al.* (2011 ▸). The AuNP diameter was determined by TEM as *d*_core_ = 27.7 ± 2.1 nm. The ligand α-methoxypoly(ethylene glycol)-ω-(11-mercaptoundecanoate) (PEGMUA) with a molecular weight of approximately 2000 g mol^−1^ and the AuNP coated with PEGMUA were synthesized, purified and characterized as described previously (Schulz *et al.*, 2013 ▸, 2016 ▸). The synthesis yields stable AuNP–PEGMUA conjugates in aqueous solution without significant amounts of free ligand. The concentrations were adjusted by repeated centrifugation and dilution. The AuNP concentrations were determined by the absorbance of the AuNP–PEGMUA solutions at λ = 450 nm, as described by Haiss *et al.* (2007 ▸). In general, such AuNP–PEGMUA systems are very stable and can be dried by reversible self-assembly, *i.e.* dried clusters can be redispersed in a variety of solvents.

AuNP–PEGMUA films were prepared on square silicon nitride membranes in square silicon supporting frames (Silson Ltd, UK). The membrane area was 3.0 × 3.0 mm with a thickness of 1 µm. The frame size was 7.5 × 7.5 mm and the frame thickness was 535 µm. Under these conditions, the AuNP–PEGMUA solutions dry in a well defined volume and the film thickness can be controlled by the AuNP concentration. For each film, 7.5 µl of AuNP–PEGMUA was pipetted onto the framed membrane and dried at *T* = 65°C. Two example concentrations were investigated that yielded different average sample thicknesses; the thick sample was prepared with 400 n*M* and the thin sample with 200 n*M* AuNP–PEGMUA.

### 2.2. Scanning electron microscopy   

The AuNP films were imaged with a LEO 1550 (Carl Zeiss, Germany) field-emission scanning electron microscope (SEM) operating at 20 kV accelerating voltage. To estimate the film thickness (see supporting information), the SiN membranes were fixed onto self-adhesive carbon pads and the silicon frame was removed; this procedure destroys the SiN membranes.

### 2.3. Coherent X-ray scattering   

The coherent X-ray scattering experiments were performed on beamline P10 at PETRA III (DESY, Hamburg) using the GINIX setup (Kalbfleisch *et al.*, 2011 ▸; Giewekemeyer *et al.*, 2013 ▸). The beam size was defined by a Kirkpatrick–Baez (KB) mirror system to 400 × 400 nm. The X-ray photon energy was set to *E* = 13.8 keV, corresponding to a wavelength of λ_X-ray_ = 0.0898 Å. The experiment was performed in small-angle X-ray scattering (SAXS) geometry with a sample-to-detector distance of 5 m. For the detector, a Dectris Eiger X 4M detector was used.

For each sample, several regions of interest (ROIs) were defined by optical microscopy which are representative of the particular sample. At these ROIs, scattering patterns were taken in grids of typically 30 × 30 µm in steps of 500 nm in the *x* and *y* directions. The exposure time was set to 1 s per pattern. Afterwards, the patterns were corrected for contributions from the SiN membranes and analysed following the XCCA scheme discussed elsewhere (Lehmkühler *et al.*, 2014 ▸; Schroer *et al.*, 2015 ▸).

### 2.4. X-ray cross-correlation analysis   

In XCCA experiments, the orientational order within a sample is probed *via* angular correlation functions. In the case of a wavevector transfer of constant modulus *q* = |**q**| = [4πsin(θ/2)/λ], where θ denotes the scattering angle, the correlation function is given by (Wochner *et al.*, 2009 ▸)
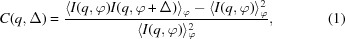
with the azimuthal angle φ, the angular difference Δ and the scattering intensity *I*(*q*,φ) = *I*(**q**). Typically, the degree and type of orientational order is quantified by the Fourier coefficient of 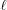
th order 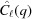 with respect to Δ (Altarelli *et al.*, 2010 ▸; Kurta *et al.*, 2012 ▸; Lehmkühler *et al.*, 2014 ▸; Schroer *et al.*, 2014 ▸, 2015 ▸) that is connected to the Fourier coefficients 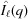 of the normalized intensity, 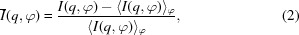

*via* the Wiener–Khinchin theorem
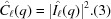
For instance, a hexagonal symmetry results in a maximum of 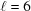 (Altarelli *et al.*, 2010 ▸; Lehmkühler *et al.*, 2014 ▸). Thus, information on the orientational order in the sample can be obtained by the angular Fourier transform of the scattering pattern
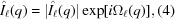
where 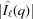 is a measure of strength for the corresponding symmetry of order 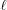 and the phase 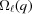 provides information about the orientation of a given symmetry of the scattering pattern that relates to the orientation of domains in the sample (Schroer *et al.*, 2015 ▸). In order to get access to the overall sample structure, the variance, which is given by
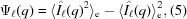
was demonstrated to be an appropriate ensemble average (Lehmkühler *et al.*, 2014 ▸); 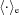 denotes an ensemble average over all realizations of the sample, here referring to measured spots of the sample.

In order to obtain information on the sample structure that goes beyond static structure in XCCA experiments, some experimental conditions have to be fulfilled. For instance, the appearance of odd symmetries has been discussed in previous studies and may originate from the curvature of the Ewald sphere as well as experimental limitations and noise (Lehmkühler *et al.*, 2014 ▸; Schroer *et al.*, 2014 ▸; Liu *et al.*, 2016 ▸). In our study, these effects are reduced because of the SAXS geometry and the use of thin samples with a thickness below 1 µm.

## 3. Results and discussion   

As a result of the different concentrations of particles in the AuNP–PEGMUA solutions, the thick sample corresponds to a rather thick layer of gold particles (*d*_1_ = 460 ± 180 nm, see supporting information) resulting in high scattering intensities and the thin sample consists of a thin layer (*d*_2_ = 190 ± 90 nm) with lower scattering intensities.

### 3.1. SEM and SAXS characterization   

**Figure 1:**
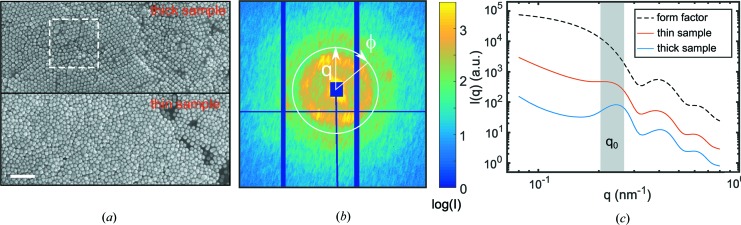
(*a*) SEM images from ROIs of both samples. The scale bar corresponds to 200 nm. The dashed square in the top part of the figure marks the beam dimensions. (*b*) Example scattering pattern from the thick sample, with definitions of *q* and the azimuthal angle φ. (*c*) Averaged *I*(*q*) for the thin and thick samples 1 and 2. The grey area highlights the region of measured *q*_0_, see also Figs. 2 ▸(*c*) and 2 ▸(*d*). A calculated form factor is shown for comparison. For clarity, the curves are offset.

SEM images from ROIs of both samples are shown in Fig. 1 ▸(*a*). For the thick sample, the left part is characterized by long-range hexagonal crystal-like order. In contrast, the right part is dominated by square-like order on shorter length scales. The thin sample has more amorphous order with only local patches of hexagonal or square symmetry. However, imaging techniques such as SEM only provide access to the surface layer of the sample so that conclusions on the sample structure are limited. Here, X-ray scattering provides information about the structure of the total exposed sample volume. A typical region illuminated by the X-ray beam is displayed in Fig. 1 ▸(*a*), consisting of lateral layers of about 15 × 15 particles exposed to the beam. Thus, on average about 4000 particles for the thick sample and 1600 particles for the thin sample give rise to a single diffraction pattern, allowing us to measure the three-dimensional structure of the sample that is still on a local scale. It is important to note that, for thin samples, the absorption of X-rays can be neglected and the intensity in the SAXS regime becomes 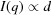, where *d* is the sample thickness. A distribution of *I*(*q*) from all sample spots measured can be found in the supporting information.

A scattering pattern from the thick sample is shown in Fig. 1 ▸(*b*) and the intensity *I*(*q*) averaged over all measured sample spots is shown in Fig. 1 ▸(*c*) for both samples. At large *q*, the signal is dominated by the form factor *P*(*q*) of the particles, which is the same for all samples studied. *P*(*q*) can be modelled by a form factor of polydisperse spheres with an average particle radius of 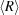 = 13.65 nm ± 0.05 nm and a size polydispersity of 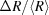 = 11%, which is in agreement with electron microscopy analyses. Around 0.20–0.26 nm^−1^ (marked by the grey area and labelled *q*_0_) a peak is visible. The intensity in SAXS experiments of spherical particles with low size polydispersity is given by 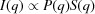 (de Jeu, 2016 ▸), with the structure factor *S*(*q*) as the Fourier transform of the pair distribution function *g*(*r*). Hence, the interparticle interference can be associated with the next-neighbour distance, causing a peak in *S*(*q*) at *q*_0_. For the thick sample, the peak is well developed, possibly a result of the higher degree of order compared with the thin sample. Ordering is also indicated by the modulations on rings of constant *q* in Fig. 1 ▸(*b*) that may originate from Bragg reflections as a result of crystalline order.

**Figure 2:**
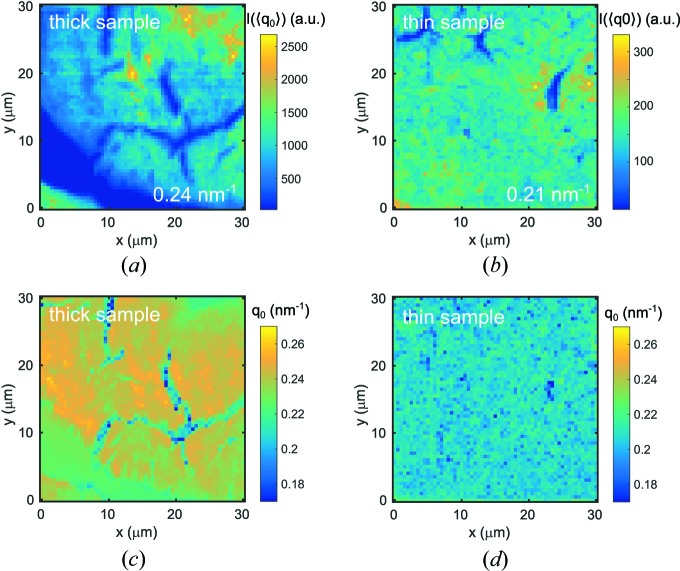
(*a*) and (*b*) Scanning intensity maps for both samples at 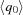. The average 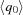 studied is indicated in the colour scales. (*c*) and (*d*) Scanning maps of *q*_0_.

The spatial distribution of intensity at the position of the structure factor peak 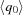 is displayed in Figs. 2 ▸(*a*) and 2 ▸(*b*). Here, 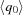 was chosen as the average over the whole map studied, see Fig. 1 ▸(*c*). The smaller value for the thin sample of 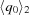 = 0.21 nm^−1^ compared with 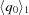 = 0.24 nm^−1^ for the thick sample suggests a closer packing of particles in the thick sample. *I*(*q*_0_) of the thick sample is governed by the appearance of ‘hot spots’ of high scattering intensity in the top right section and cracks throughout the sample area, whereas the thin sample appears to be more homogeneous. The position of *q*_0_ was obtained by a fit of *I*(*q*) in the vicinity of the peak and found to differ slightly for both samples over the studied area, as demonstrated in Figs. 2 ▸(*c*) and 2 ▸(*d*). Likewise, variations can be found for the thick sample between film cracks and homogeneous regions. Interestingly, the weak scattering signal in the region in the bottom left of the studied area is characterized by a rather constant *q*_0_, suggesting a very homogeneous thin film.

### 3.2. Average orientational order   

**Figure 3:**
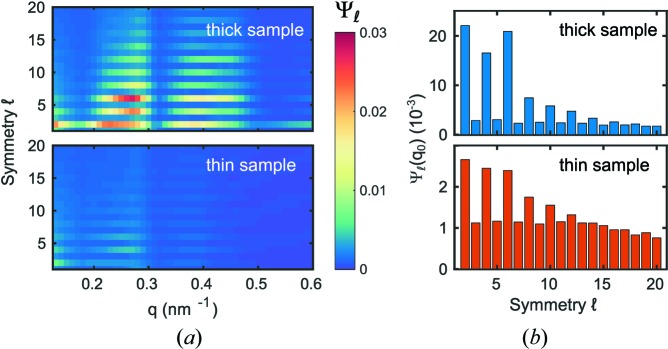
(*a*) Ensemble averaged angular correlation functions 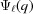 for the thick sample (top) and the thin sample (bottom). (*b*) 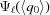, *i.e.* at the structure factor peak.

To quantify the overall orientational order in both samples, the ensemble average 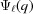 is calculated following equation (5). The results are shown in Fig. 3 ▸(*a*) for 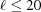. Similar to studies of colloidal crystals (Lehmkühler *et al.*, 2016 ▸) the even coefficients peak because of Friedel’s law, *i.e.* *I*(−**q**) = *I*(**q**). This is also the reason for the appearance of peaks at 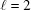 (Wochner *et al.*, 2009 ▸). Furthermore, coefficients 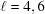 dominate around *q*_0_ and are less pronounced around *q* ≃ 0.4 nm^−1^, *i.e.* in the vicinity of the second structure factor peak (Schroer, Schulz *et al.*, 2016 ▸). These can be connected to the dominant cubic and hexagonal local order as observed in the SEM images in Fig. 1 ▸(*a*). Comparing the two samples, the thinner sample shows, by a factor of about ten, less pronounced orientational order. This is highlighted in Fig. 3 ▸(*b*) where 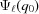 is shown for both samples. As seen for the thick sample, two-, four- and sixfold symmetries dominate for the thin sample. Furthermore, odd coefficients can be observed of the order of 10^−3^, which we assign to the background level that reduces slightly for large 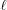.

### 3.3. Maps of orientational order   

**Figure 4:**
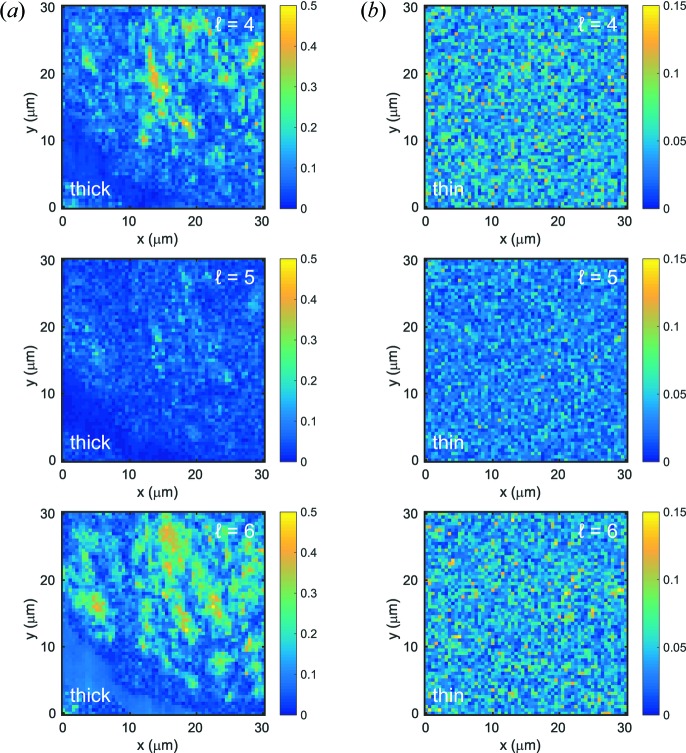
Spatial maps of the degree of orientational order 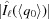 with 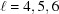, as indicated for (*a*) the thick sample and (*b*) the thin sample. Note the different colour scales for panels (*a*) and (*b*). The maps correspond to the same data shown in Fig. 2 ▸.

Fourier coefficients of intensity 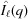 were calculated at each measured spot. Since four- and sixfold symmetries dominate the local orientational order, we focus on 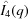 and 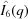. In addition, we choose 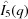 as a measure for a symmetry that is not connected to any dominant order, thus representing a background signal. The resulting maps of the degree of orientational order 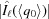 are shown in Fig. 4 ▸ for 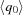. For the thin sample, the maps are homogeneous and differ slightly in amplitude, indicating a rather homogeneous local order. In contrast, the thick sample shows a more complex variation in orientational order. Regions of strong four- and sixfold symmetry can be identified, *e.g.* around *x* = 15 µm and *y* = 20 µm for 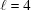, which is also characterized by a strong scattering intensity, see Fig. 2 ▸(*a*). In general, the six- and fourfold symmetries are not spatially correlated, indicating a heterogeneous structure of the sample with hexagonal and cubic local order. Therein, hexagonal order appears to be more frequent and stronger. Most importantly, the 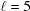 coefficient is weak and homogeneous throughout the maps, suggesting that the assumption of a background with no structural information is justified. We do not see any strong correlation between intensity *I*(*q*) and local order 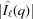, similar to what occurs in silica particle films where local crystal spots were observed (Schroer *et al.*, 2015 ▸).

**Figure 5:**
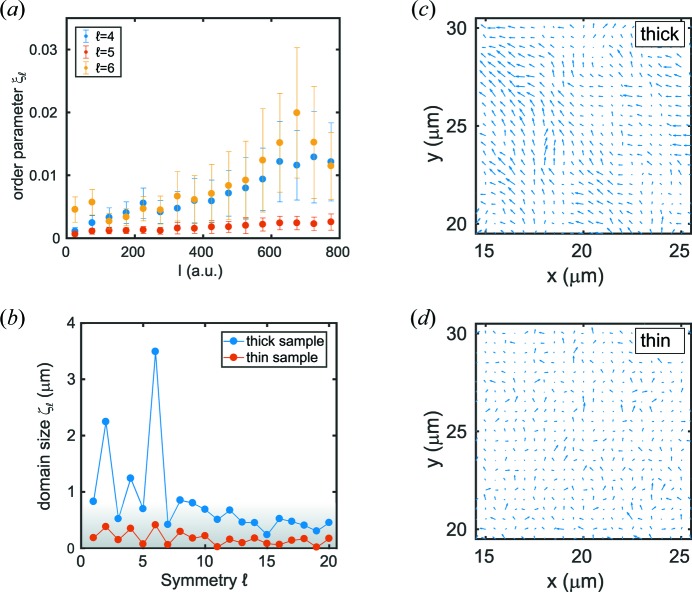
(*a*) Order parameter 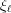 as function of intensity for 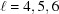, averaged over all nine ROIs of both samples. (*b*) Domain size 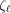. The grey area marks domain sizes less than the possible resolution of the step size of 0.5 µm. (*c*) and (*d*) Orientation of ordered patches of 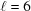. The length of the arrows is given by the degree of order 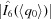 and its orientation by the phase 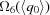.

The degree of local structure observed in the spatial maps suggests a strong dependence of local order on the film thickness. This aspect is analysed in more detail in Fig. 5 ▸(*a*). Here, the order parameter 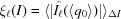 is shown as a function of intensity, *i.e.* sample thickness. It is given by 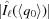, averaged over bins of intensity 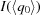 with width Δ*I* = 50 a.u. All nine studied ROIs of the two samples were used in the calculation of 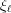, in total >36 000 scattering patterns. The error bars represent the standard deviation of all data within intensity bins of width Δ*I*. As discussed, the Fourier modes that do not reflect a certain local order, in this case 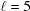, do not depend on the sample thickness and represent a background signal with small variations. In contrast, the Fourier modes 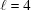 and 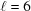 that reflect cubic and hexagonal order increase with intensity and thus film thickness. Furthermore, their variation as reflected by the error bars also increases. This is a consequence of the heterogeneous nature of the self-assembled patches of local order. For instance, a rather thick sample [high 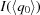] with hexagonal local order leads to a large 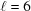 and weak 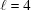 symmetry and *vice versa*, resulting in a large spread of coefficients at a given intensity, *i.e.* film thickness.

In addition to this variation in the degree of local order, the spatial maps in Fig. 2 ▸ also suggest the formation of patches of preferred local order, similar to observations in binary silica colloids (Schroer *et al.*, 2015 ▸). In order to determine typical patch sizes, spatial autocorrelation functions of the symmetry orientations are calculated at *q*_0_,
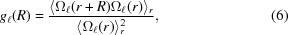
where *r* = (Δ*x* ^2^ + Δ*y* ^2^)^1/2^ denotes the distance between two sample spots. We note that the thin sample regime in the bottom left of the thick sample was not taken into account for the calculation of 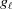. The corresponding correlation functions are modelled by Lorentzian functions, where the FWHM is a measure of the corresponding domain size 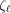 of the local order of symmetry 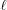. The resulting 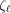 is shown in Fig. 5 ▸(*b*) for both samples and 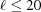. Fourier modes that evidently do not reflect any orientational order show very small or non-detectable domain sizes (*i.e.* below 0.5 µm), such as for the thin sample and for 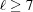 for the thick sample, while for hexagonally ordered structures in the thick sample we find the largest domains of 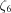 ≃ 3.5 µm. These differences are highlighted in Figs. 5 ▸(*c*) and 5 ▸(*d*), showing the degree and orientation of sixfold symmetry in selected areas of both samples. The similarity of neighbouring arrows with respect to length and orientation for the thick sample visualizes the results of larger domain sizes compared with the thin sample, where domain sizes exceeding the scanning step size of 0.5 µm cannot be observed.

## 4. Summary and conclusions   

**Figure 6:**
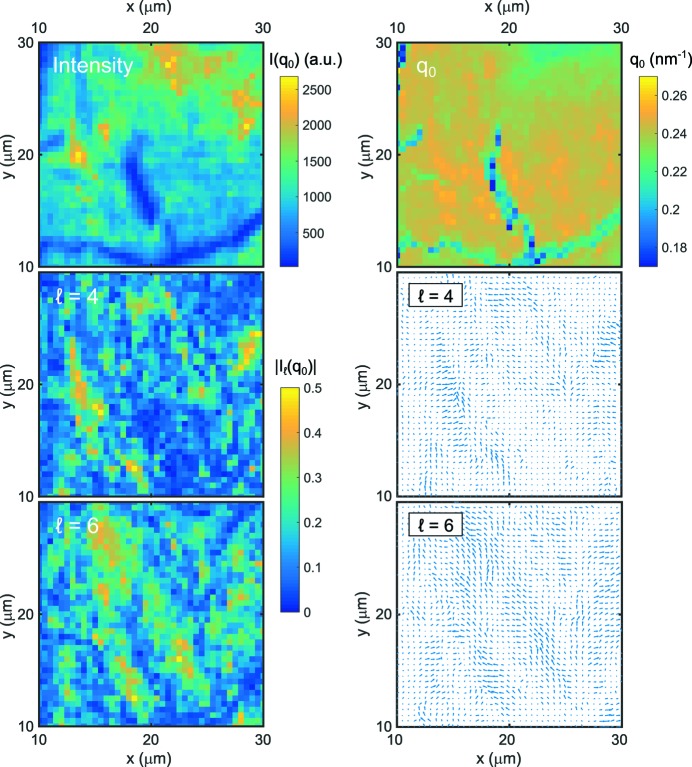
Results of the thick sample for 10 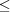 *x* 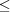 30 µm and 10 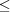 *y* 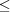 30 µm. Spatial maps of intensity, *q*_0_, 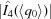, 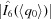, 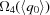 and 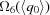 are compared.

In summary, we have investigated self-assembled films of AuNP coated with PEGMUA by means of XCCA. We observed structurally heterogeneous films that are characterized by (i) dominant four- and sixfold symmetries and (ii) patch sizes of 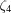 ≃ 1 µm and 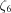 ≃ 3.5 µm for cubic and hexagonal order, respectively. The observed heterogeneous structure of the thick sample is summarized in Fig. 6 ▸. Therein, sections (10 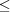 *x* 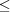 30 µm and 10 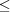 *y* 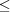 30 µm) of the intensity and *q*_0_ maps from Fig. 2 ▸ are compared directly with the spatial maps of four- and sixfold symmetry as well as the corresponding orientations. Results from different spots of the samples can be found in the supporting information. Although some hot spots of intensity appeared to be correlated with a high degree of fourfold symmetry (*e.g.* for *x* ≃ 13 and *y* ≃ 20 µm), other regions did not show any correlation. This is even more pronounced for 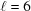, where a high value of 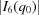 is not necessarily reflected by high intensities.

The average degree of four- and sixfold order increases as a function of the illuminated sample volume with a wide spread of the corresponding order parameters 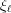. This suggests that a specific minimum amount of sample is necessary to form well ordered local structures. Furthermore, the increasing variation in 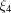 and 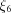 with sample thickness demonstrates the high degree of structural heterogeneity of ordered domains for larger film thicknesses. Most importantly, all other symmetries are thickness independent, and their patch sizes are very small and mostly below the threshold of 500 nm, emphasizing the lack of further dominant local structure apart from 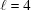 and 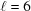. This information cannot be obtained by electron microscopy, as demonstrated with complementary SEM measurements, and it is highly valuable for understanding the structure and properties of complex self-assembled superstructures.

## Supplementary Material

Additional figures. DOI: 10.1107/S2052252518005407/ro5011sup1.pdf
