# Supplementary material for: Heterogeneous local order in self-assembled nanoparticle films revealed by X-ray cross-correlations
Source: IUCrJ. 2018 Apr 28;5(Pt 3):354–60. doi: 10.1107/S2052252518005407 (PMC5929381; doi:10.1107/S2052252518005407)
Supplement: Supplementary file 1 [file m-05-00354-sup1.pdf]

IUCrJ

Volume 5 (2018)

Supporting information for article:

Heterogeneous local order in self-assembled nanoparticle films revealed by X-ray cross-correlations

Felix Lehmkühler, Florian Schulz, Martin A. Schroer, Lara Frenzel, Holger Lange and Gerhard Grübel

Heterogeneous local order in self-assembled nanoparticle films revealed by X-ray cross correlations – Supporting Information

Felix Lehmkühler, Florian Schulz, Martin A. Schroer, Lara Frenzel, Holger Lange, and Gerhard Grübel

In this supporting information, we present further SEM images taken from both samples, XCCA results from addition sample regions as well as distributions of intensity which is a measure of sample thickness.

SEM images – overview

Thick Sample

In Fig. S1, overview images are shown for sample 1 at two different magnifications. The formation of cracks due to the drying process are clearly visible.

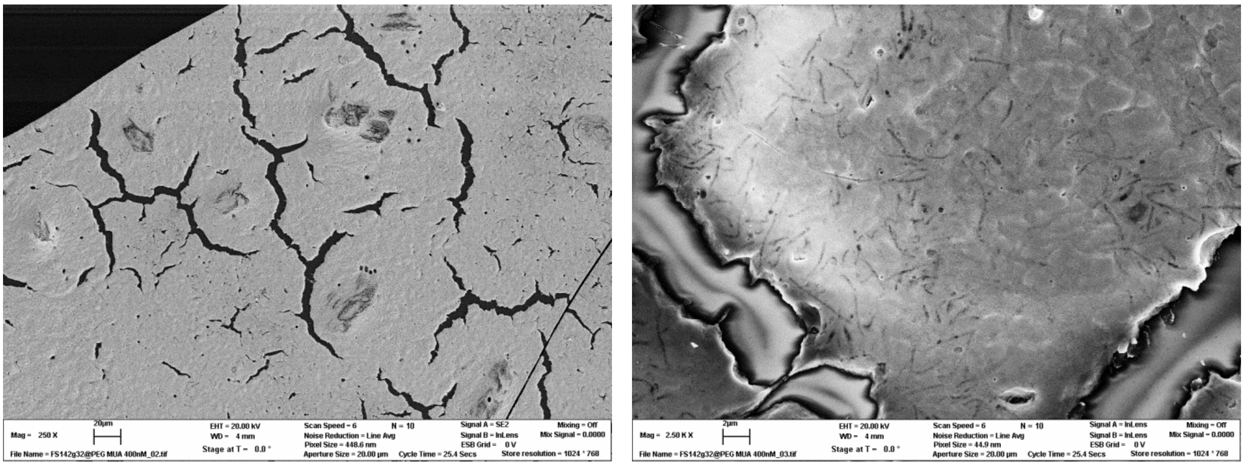

Figure S1: SEM images from the thick sample. The length scale is given by the bar in the figure legend.

Sample 2

Similar structures can be found in the overview SEM image for the thin sample shown in Fig. S2.

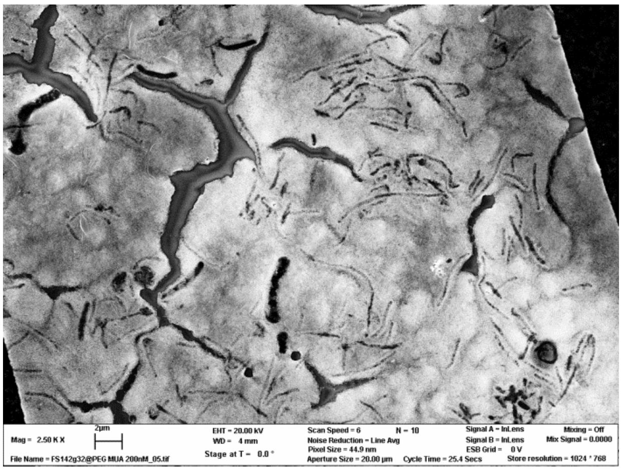

Figure S2: SEM image from the thin sample. The length scale is given by the bar in the figure legend.

SEM images - thickness evaluation

In order to estimate the thickness of the colloidal films, SEM image were taken from films where the silicon nitrite membrane has been removed. Multiple images have been taken for both sample, examples are shown in Fig. S3 for sample 1 and Fig. S4 for sample 2, respectively. From the analysis of these SEM patterns, we estimate the film thicknesses to $d_1 = 460 \pm 180$ nm for the thick sample and $d_2 = 190 \pm 90$ nm for the thin sample.

Thick Sample

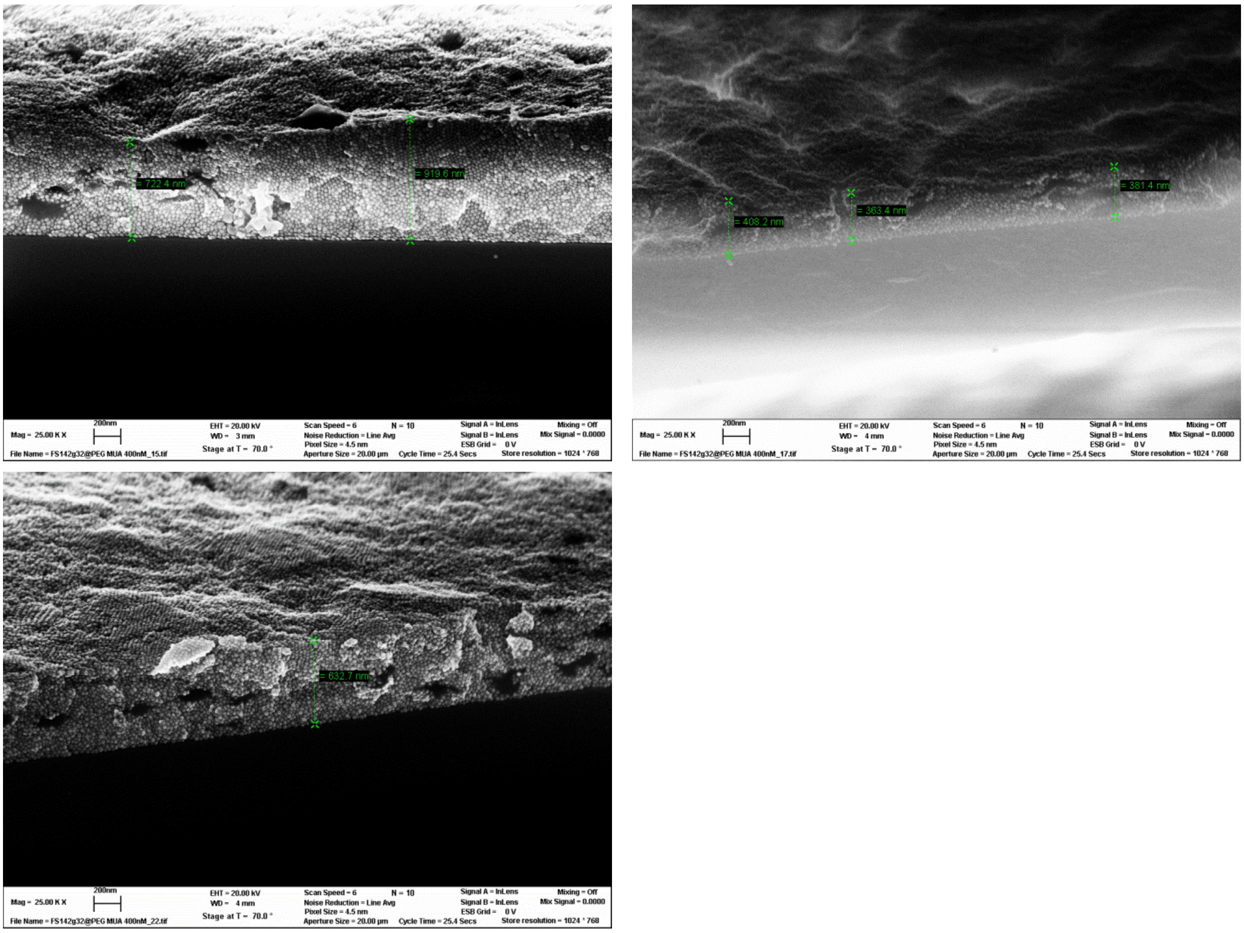

Figure S3: Exemplary cuts of the thick sample for thickness estimation by SEM.

Thin Sample

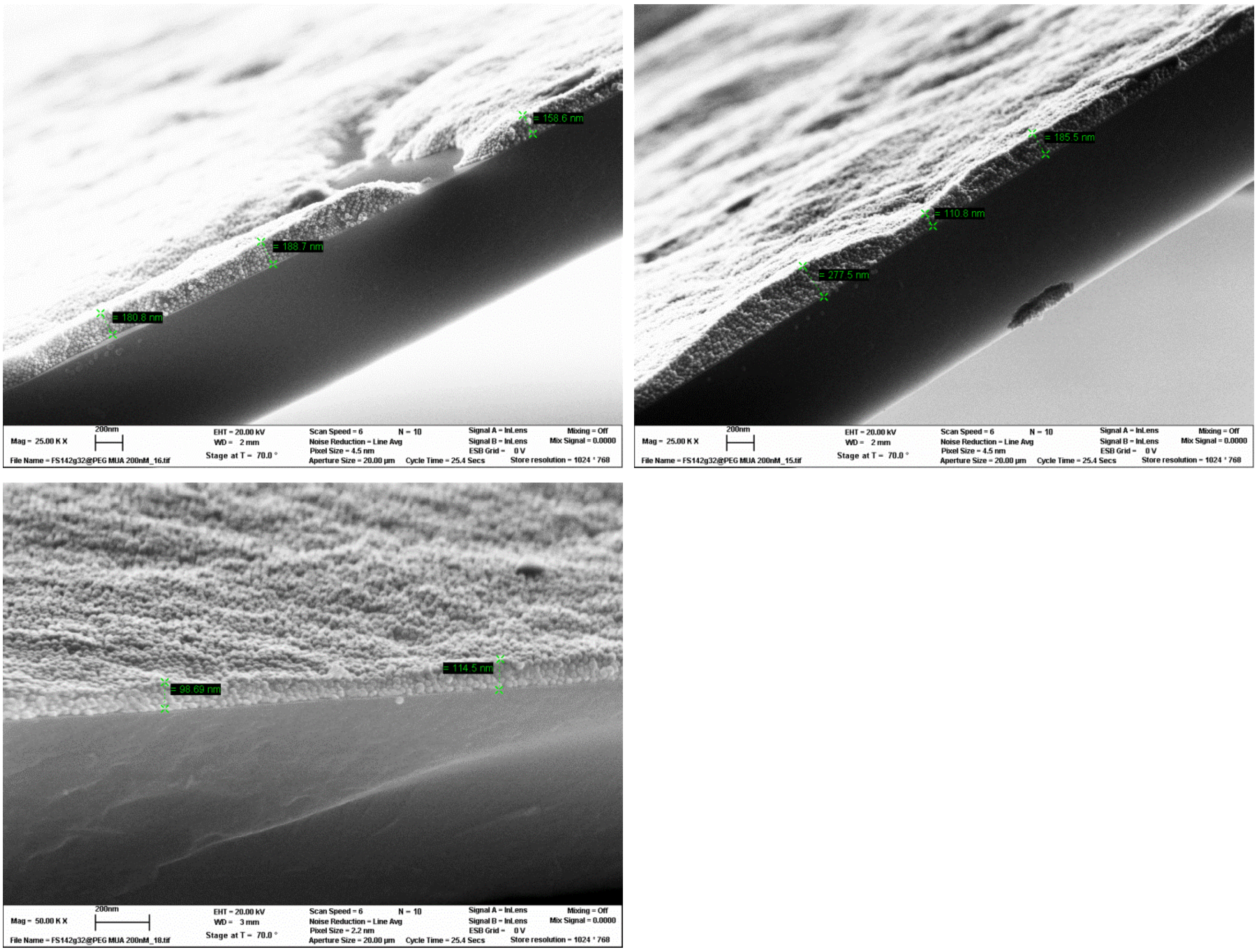

Figure S4: Exemplary cuts of the thin sample for thickness estimation by SEM.

XCCA results from additional regions

The following figures S5 to S8 show two more regions per sample. Therein, the intensity maps are shown in top left, the q_0 maps in bottom left. On the right panel, the orientational order maps are shown for $\ell = 4$ and $\ell = 6$. Similar to the region discussed in the paper, different structure such das crack and rings are visible, characterized by a heterogeneous local order.

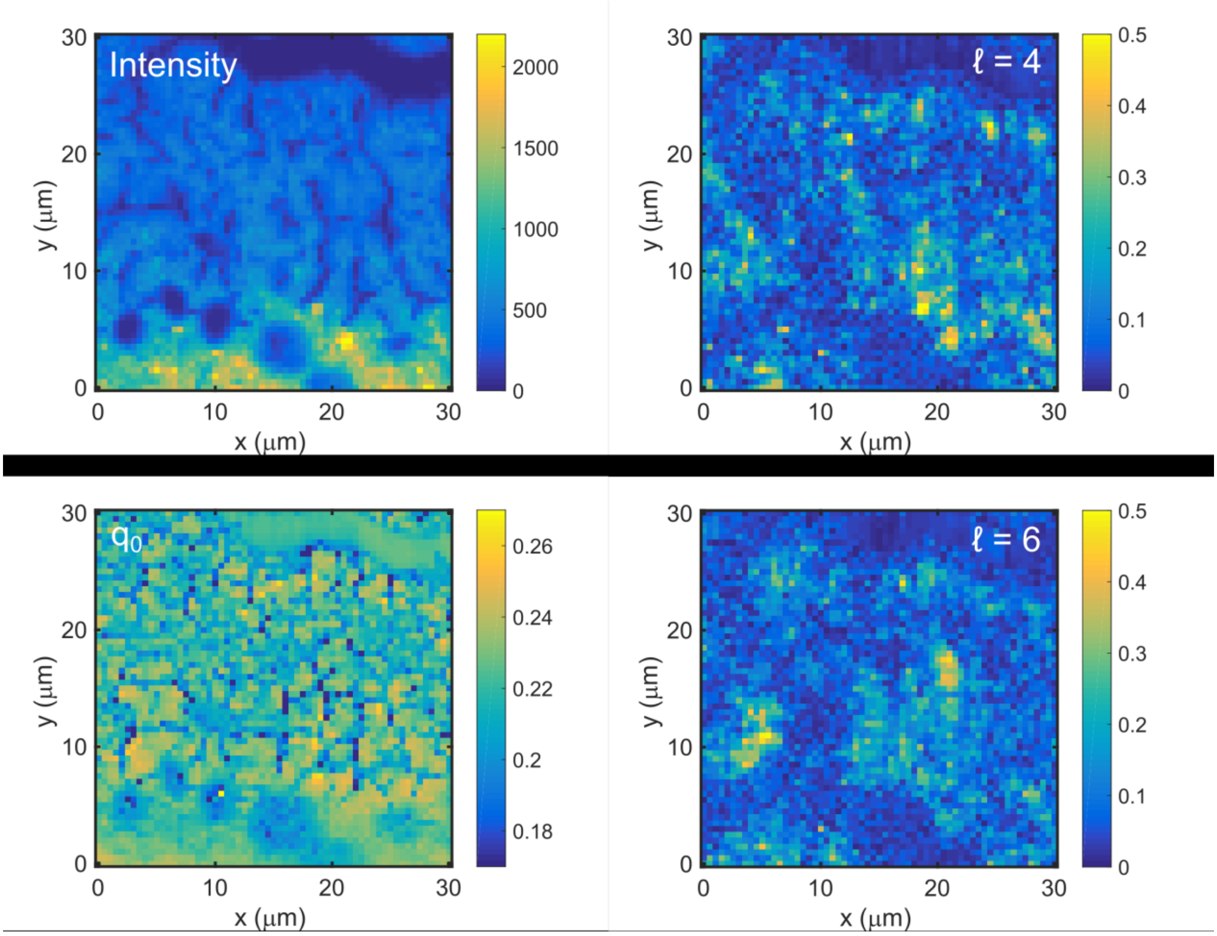

Figure S5: Maps of intensity, q_0 and orientational order ($\ell = 4, \ell = 6$) from a region of the thick sample. The top part of the sample region is dominated by small cracks. The local order is found to show heterogeneous regions of 4- and 6-fold symmetry.

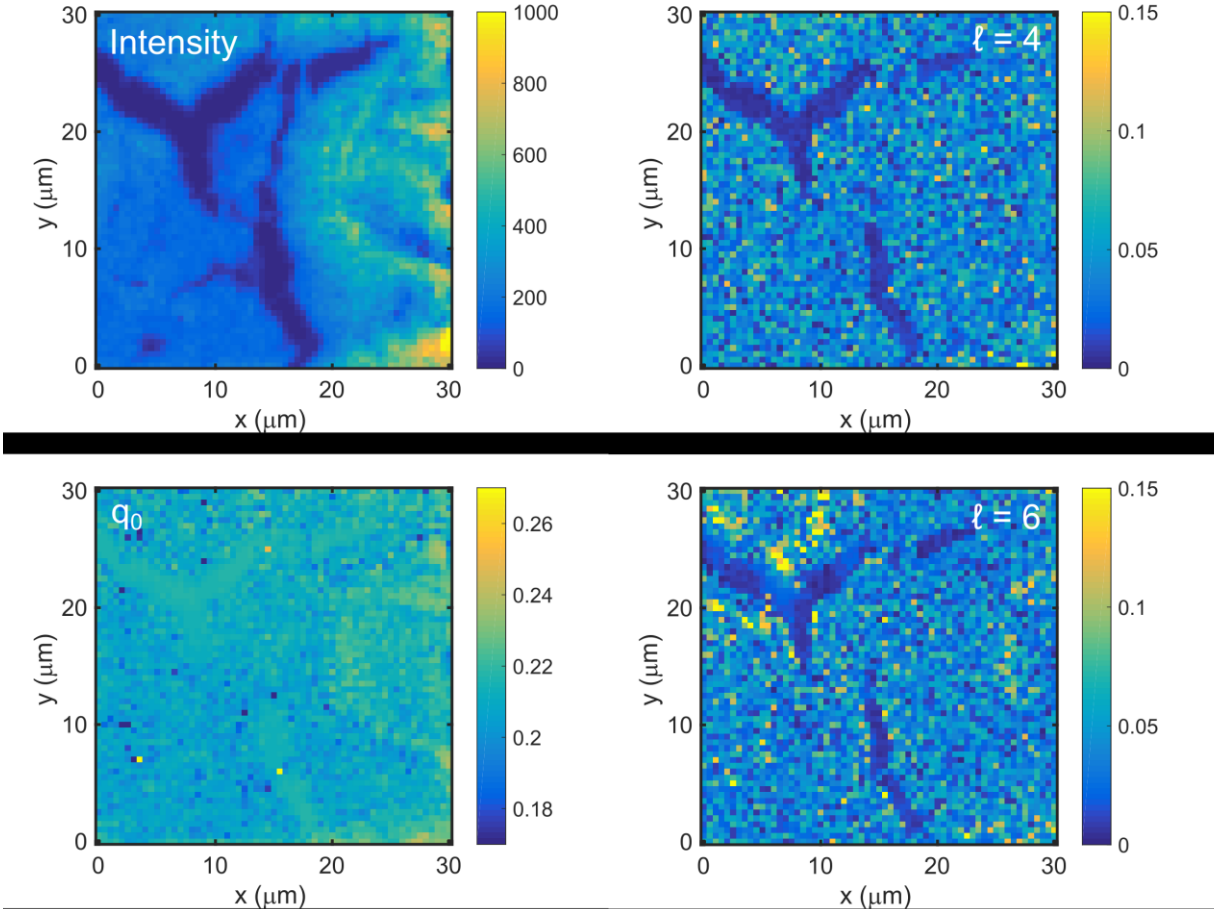

Figure S6: Maps of intensity, q_0 and orientational order ($\ell = 4, \ell = 6$) from a region of the thick sample. This region represents a rather thin part, reflected by low values of q_0 and low degree of orientational order.

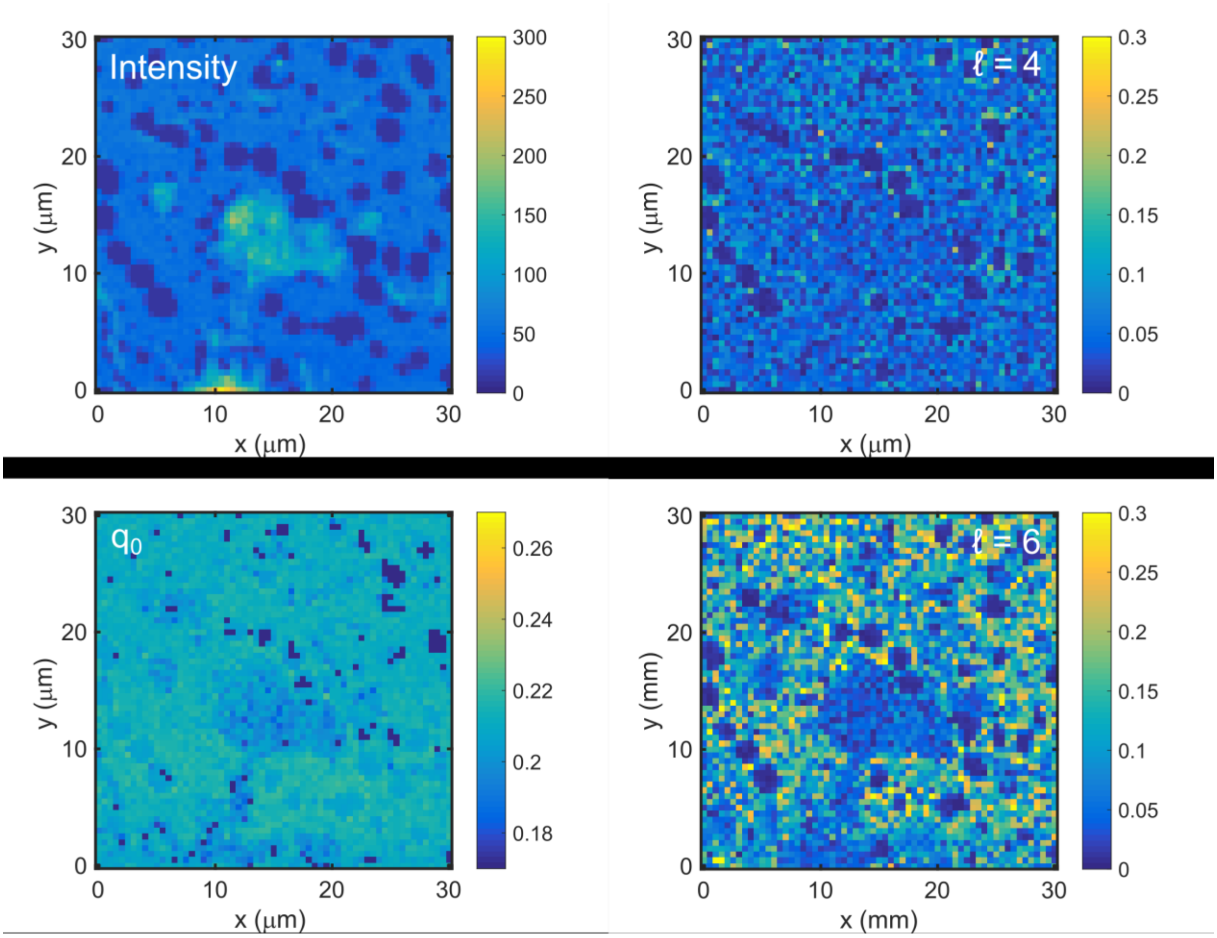

Figure S7: Maps of intensity, q_0 and orientational order ($\ell = 4, \ell = 6$) from a region of the thin sample. This region is characterized by a thin layer with empty spots. Nevertheless, the edges of the empty spots show a high 6-fold symmetry.

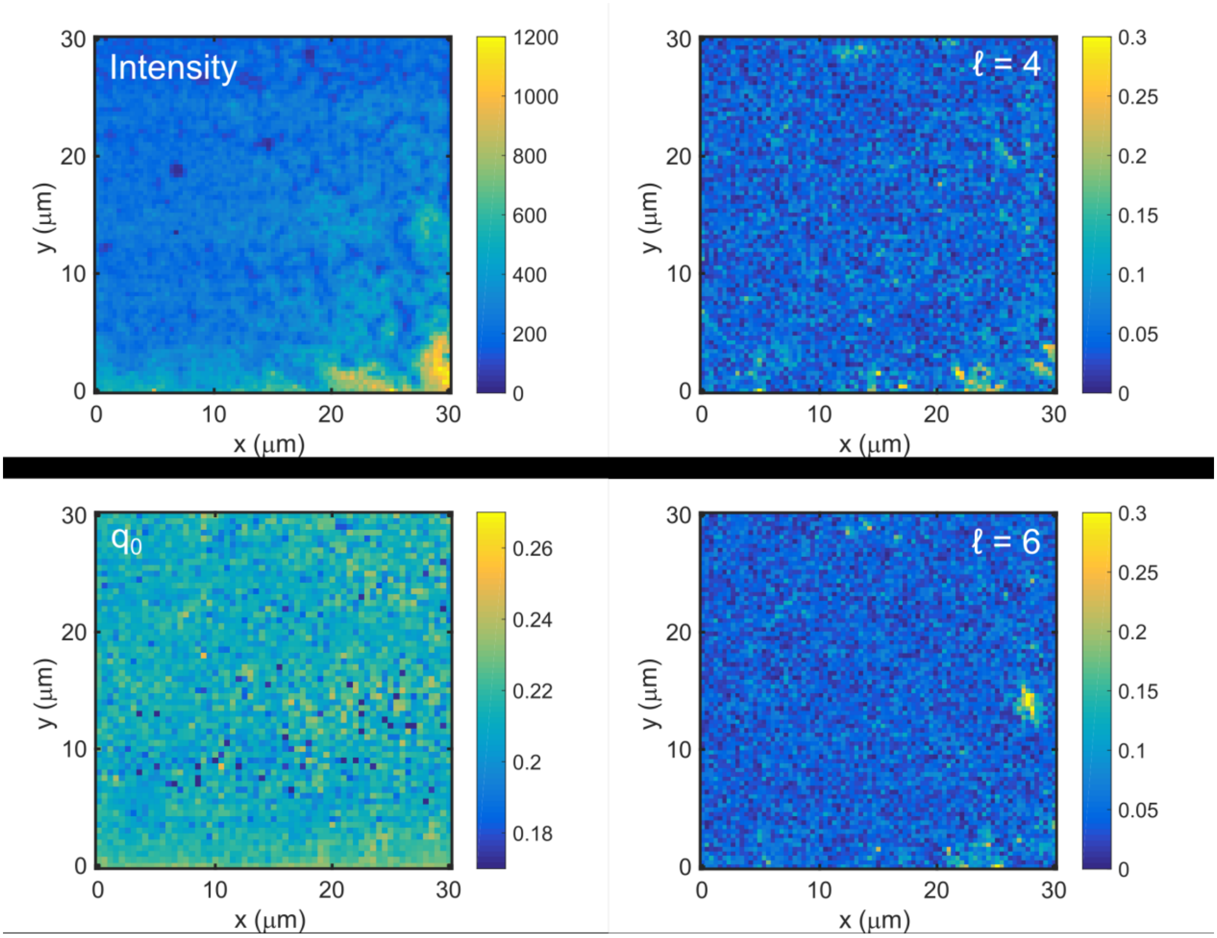

Figure S8: Maps of intensity, q_0 and orientational order ($\ell = 4, \ell = 6$) from a region of the thick sample. This sample is homegeneous whith a thick part at the bottom right.

Thickness distribution

The thickness of the sample at the measured spots is estimated from the scattered intensity. Therefore, we show a histogram of intensity in Figure S9. The blue and red lines correspond to the two sample regions described in the paper, respectively. The yellow line is the histogram of all studied sample spots (>36000).

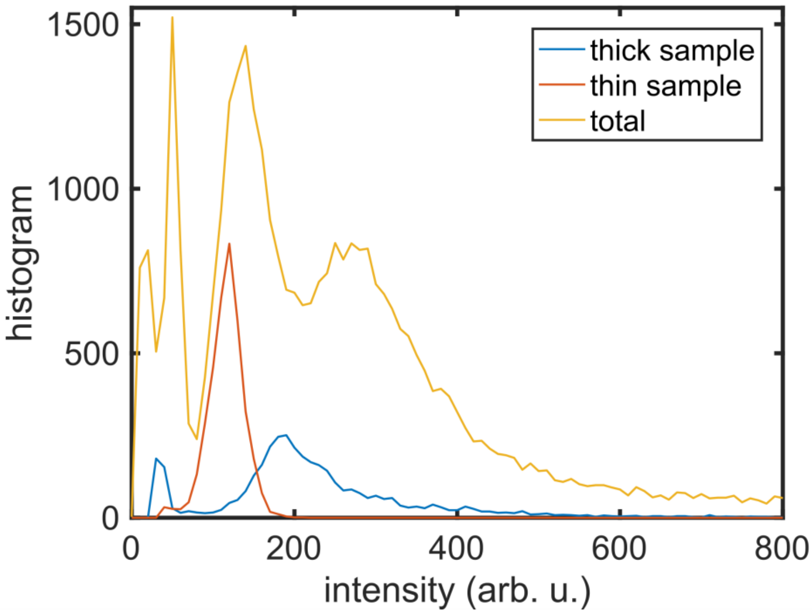

Figure S9: Intensity histograms for the thick and thin sample, as well as all studied samples.
